# A joint model of regulatory and metabolic networks

**DOI:** 10.1186/1471-2105-7-332

**Published:** 2006-07-04

**Authors:** Chen-Hsiang Yeang, Martin Vingron

**Affiliations:** 1 Center for Biomolecular Science & Engineering, University of California, Santa Cruz, 1156 High Street, Santa Cruz, CA 95064, USA; 2 Max-Planck Institute for Molecular Genetics, 73 Ihnerstraße, Berlin, Germany

## Abstract

**Background:**

Gene regulation and metabolic reactions are two primary activities of life. Although many works have been dedicated to study each system, the coupling between them is less well understood. To bridge this gap, we propose a joint model of gene regulation and metabolic reactions.

**Results:**

We integrate regulatory and metabolic networks by adding links specifying the feedback control from the substrates of metabolic reactions to enzyme gene expressions. We adopt two alternative approaches to build those links: inferring the links between metabolites and transcription factors to fit the data or explicitly encoding the general hypotheses of feedback control as links between metabolites and enzyme expressions. A perturbation data is explained by paths in the joint network if the predicted response along the paths is consistent with the observed response. The consistency requirement for explaining the perturbation data imposes constraints on the attributes in the network such as the functions of links and the activities of paths. We build a probabilistic graphical model over the attributes to specify these constraints, and apply an inference algorithm to identify the attribute values which optimally explain the data. The inferred models allow us to 1) identify the feedback links between metabolites and regulators and their functions, 2) identify the active paths responsible for relaying perturbation effects, 3) computationally test the general hypotheses pertaining to the feedback control of enzyme expressions, 4) evaluate the advantage of an integrated model over separate systems.

**Conclusion:**

The modeling results provide insight about the mechanisms of the coupling between the two systems and possible "design rules" pertaining to enzyme gene regulation. The model can be used to investigate the less well-probed systems and generate consistent hypotheses and predictions for further validation.

## Background

Gene regulation and metabolic reactions are two primary activities of life. Despite vast amount of works have been dedicated to study each system, the coupling between the two systems is relatively less probed. It is therefore necessary to shift focus from individual systems to their integration.

The coupling between metabolic reactions and gene regulation is supported by abundant direct and indirect evidence. Some assays indicate substrates of metabolic reactions influence the activities of transcription factors or signal transduction pathways (e.g., [1-4]). More studies show metabolic enzymes are differentially expressed under different nutrient conditions or enzyme knock-outs (e.g., [5-9]). These studies suggest 1) metabolic reactions are controlled by enzyme quantities besides concentrations of substrates and allosteric regulation of enzymes, 2) enzyme gene expressions are indirectly regulated by metabolites. Both implications are sensible from an evolutionary perspective. Despite its slowness, gene regulation yields a persistent response which can shift the cell from one metabolic "mode" to another. It is also easier to control due to the modularity of gene regulation and the separation between functional (exons) and regulatory (promoters) elements in the genome. The influence of metabolites on enzyme gene regulation is a necessary arrangement to make the systems (both metabolic and regulatory) responsive to environmental changes and maintain the homeostasis of metabolism.

Previous methods of modeling the joint processes of gene regulation and metabolic reactions fall into three general categories. The first approach explicitly establishes the links between specific metabolites and transcription factors and models the influence between metabolites and gene expression. Examples include imposing binary constraints from gene expression data on metabolic fluxes [10,11], explaining mRNA and protein expression data under different carbon sources with cascades of protein interactions [12], and incorporating (metabolite, regulator) links and functions in a Boolean network [13]. While this approach can model the mutual influence between metabolites and transcription factors, existing works tend to focus on either direction. [10,11] emphasize the constraints of enzyme expression levels on metabolic fluxes, and [12,13] focus on the effect of metabolites on gene expression. Moreover, although these models in principle can learn the interactions between metabolites and transcription factors, most current works extract these links from previous studies and treat them as given. The second approach integrates the two systems by proposing high-level hypotheses regarding the regulation of metabolic enzymes and validates these hypotheses by experimental data. Common hypotheses include efficient allocation of resources to optimize growth [14], combination of flexibility of flux modes and efficiency for biomass growth [15], and co-regulation of enzymes along metabolic pathways [16,17]. While these hypotheses provide insight about the design rules of the regulatory and metabolic systems, they also lack the information about the underlying mechanisms. The third approach builds a dynamic system of metabolic reactions and gene regulation. Examples include models of galactose switch [18], pheromone response pathways [19], and general metabolic reactions [20]. While dynamic systems can in principle capture the complex system behavior, they also suffer from the lack of data and unknown kinetic parameter values.

**Figure 1:**
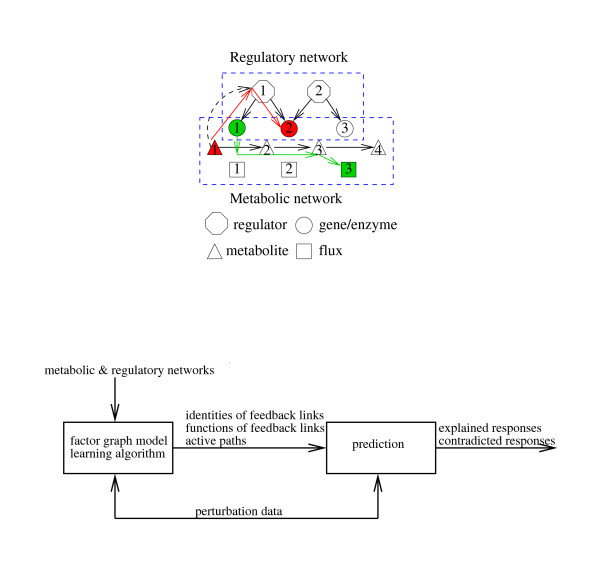
**The modeling concept and framework**. (a) A feedback link from metabolite 1 to regulator 1 is established (black dash arc). Two perturbation responses are explained by the model. The up regulation of gene 2 by supplying metabolite 1 is explained by the path marked by red arrows (metabolite 1 → regulator 1 → gene 2). The decrease of flux 3 by deleting enzyme (gene) 1 is explained by the path marked by green arrows (enzyme 1 → flux 1 → metabolite 2 → flux 2 → metabolite 3 → flux 3). Edges (metabolite 1, regulator 1) and (regulator 1, gene2) must be both positive or both negative in order to explain the first response. (b) The framework of the joint model. A factor graph model is constructed from metabolic and regulatory networks and perturbation data. A learning algorithm is applied to find the identities and functions of feedback links and active paths. These information are used to predict each perturbation response. By comparing with the observed data, each response is explained or contradicted with the prediction.

In this work we propose a joint model of gene regulation and metabolic reactions that combine the merits of the first two approaches and compensate their shortcomings. Our model aims for answering the following questions. First, we want to demonstrate whether the coupling between the two systems is essential to explain different types of perturbation data. Second, the identities and functions of the feedback links between metabolites and regulators are often unknown. We want to find those missing links from the perturbation data. Third, we want to identify the mechanisms responsible for perturbation responses. By abstractizing these mechanisms as paths in the joint network of gene regulation and metabolic reactions, this question is translated into finding the "active paths" which explain the perturbation responses. Fourth, in addition to individual (metabolite, regulator) links we are also interested in the "design rules" regarding the architecture and functions of the feedback regulation of metabolic enzymes. We want to test whether certain hypotheses pertaining to feedback regulation can better explain the experimental data. The general concept of our model is illustrated in Figure 1(a). The networks of gene regulation (links between transcription factors and regulated genes) and metabolic reactions (associations of reactants and enzymes of each reaction) are joined by edges from metabolites to transcription factors or enzyme genes. We consider the data of gene expression or metabolic fluxes by altering genes (gene knock-outs or over-expression) or metabolites (nutrient limitation or supply). A perturbation data is explained by a set of paths connecting the perturbation source (metabolites, genes) to the response (gene expression, metabolic flux) if the predicted responses along those paths coincide with the actual response. The consistency requirement for explaining perturbation data imposes constraints on the attributes in the joint network such as the functions (signs) of (metabolite, regulator) edges. For example, to explain the up regulation of gene 2 by supplying metabolite 1 in Figure 1(a) the aggregate function of the red path must be positive.

We encode these constraints as a probabilistic graphical model – factor graph – over the network attributes, and apply a graphical model learning algorithm to identify the attribute values which optimally fit the data. To build a joint network we use two methods to establish links between metabolites and genes. The first method searches among all (metabolite, regulator) pairs and incrementally augments the joint network with the links which maximize the number of explained perturbation responses. The second method explicitly adds links according to several general hypotheses pertaining to the functions of metabolic pathways and enzymes. The modeling framework is illustrated in Figure 1(b). The inputs of the model are the joint network and the perturbation data. Once the joint network is built, we incur the learning algorithm to infer the active paths and functions of the feedback links. The outputs of the model are these inferred attribute values. Once the attribute values are inferred, we can evaluate the explanatory power of the model by counting the number of perturbation responses explained by and contradicted with the model. We can also validate the predictive power of the model by using cross validation or simulation tests. Our model applies the following simplifying assumptions. First, the links from metabolites to transcription factors or enzymes are often indirect and functional. Typically, the change of metabolic conditions is sensed by a receptor protein and propagated to transcription factors via a signal transduction pathway. We do not attempt to reconstruct this mechanism due to insufficient data of signal transduction. Second, the model concentrates on four mechanisms 1) metabolites modulate the activities of transcription factors 2) the activities and quantities of transcription factors affect enzyme gene expression 3) enzyme gene expression affects the flux of the reaction it catalyzes 4) the concentration of a reactant affects the reaction flux and its products. A path in the joint network represents a combination of these mechanisms. For example, the red path in Figure 1(a) contains mechanisms (1) and (2), while the green path contains mechanisms (3) and (4). Third, for simplification we ignore several important mechanisms such as allosteric regulation of enzyme conformation, nutrient uptake, the functions of coenzymes (e.g., NADH, NADPH) and small metabolites (e.g., *H*_2_*O*, *CO*_2_, ATP, ADP, cAMP). Fourth, we only consider a special case of the combinatorial effects of multiple transcription factors or enzymes: each factor or enzyme acts independently and the change of each factor or enzyme suffices to alter gene expression or metabolic reactions.

## Results

We first describe a joint network of gene regulation and metabolic reactions and the datasets used in this work. We then define the terminology pertaining to paths in the joint network and outline the constraint-based probabilistic graphical model over model attributes. We applied two approaches to construct the joint models of metabolic reactions and gene regulation and used them to explain the perturbation responses. The first approach learns the feedback links between metabolites and regulators which best explain the data. We investigated the change of explanatory power with respect to path length and active paths connecting perturbation sources and responses in this joint model. The second approach encodes four general hypotheses pertaining to enzyme gene regulation in the joint model. To further validate the predictive power and the consistency of the model learning algorithm, we did two validation tests. We performed three cross validation tests on the perturbation data to ensure the inferred model could accurately predict the responses in the test data. We also simulated perturbation responses from the models with hypothetical feedback links and verified that the model learning algorithm could retrieve these feedback links from simulated data.

### A joint network of gene regulation and metabolic reactions

We constructed the joint network of gene regulation and metabolic reactions of *Escherichia coli*. The regulatory part of the network contains the nodes of regulators (transcription factors), operons and genes. Regulators link to operons they bind and operons link to their member genes. The metabolic part of the network contains the nodes of metabolites, fluxes of reactions, and enzymes. Metabolites at both sides of a reaction connect to the flux of this reaction with bidirectional edges. Enzymes catalyzing a reaction connects to its flux with unidirectional edges. The regulatory and metabolic parts are coupled by two types of directed edges: edges linking genes to enzymes indicating the mapping between enzyme gene identities and functions, edges linking metabolites to regulators indicating the feedback control of metabolites on regulator activities.

Many metabolic reactions are either irreversible or have a "preferred direction" under the normal growth condition. We therefore annotate the preferred directions of reactions and specify their "input" (substrate) and "output" (product) metabolites. For instance, in reaction glucose + ATP ⇌ glucose-6-phosphate + ADP, glucose is the input and glucose-6-phosphate is the output.

For *E. coli*, the substrates and enzymes of metabolic reactions, the operons of many genes, the bindings of transcription factors on some operon promoters, and the functions of these transcription factors were downloaded from the EcoCyc database [21]. We used these information to construct metabolic and regulatory networks. As a proof-of-concept demonstration we focused on genes and reactions involved in the central carbon metabolism. We included the metabolic pathways of glycolysis, tricarboxylic acid (TCA) cycle, pentose phosphorylation, Entner-Doudoroff, acetate utilization, and several other reactions connecting these reactions. We also included the enzymes catalyzing each reaction in these pathways, operons containing these enzymes, and the transcription factors which are known to control these operons according to Ecocyc. There are 49 metabolic reactions, 46 metabolites, 75 enzymes, 20 transcription factors and 126 other genes in this subset. The joint network is shown in Figures 4, 5, 6, 7. The list of these participants is provided in Additional File 16.

### Datasets

Perturbation data of *E. coli *from 5 sources were used: the gene expression datasets under acetate supply [6], glucose and amino acid limiting conditions [8], and pyruvate kinase knockout [9]; the metabolic flux datasets under pyruvate kinase knockout [22], phosphoglucose isomerase and glucose-6-phosphate dehydrogenase knockouts [23]. Metabolic flux data capture the rates of metabolite production in certain reactions. An informative review of metabolic flux measurements can be found in [24]. Table 1 summarizes the properties of these data. Notice rows 4 and 6 are two experiments from the same reference.

We quantized expression and flux data into three levels. For expression data the ratios between the perturbation and reference conditions were reported. We quantized an expression response as significantly up or down regulated if it changes more than two folds in either direction. The only exception is the expression data of pykF knock-out [9]: 1.2 fold or higher for up regulation and 0.9 fold or lower as down regulation. For flux data the percentage of fluxes relative to glucose uptake was reported. We quantized a flux response as significantly increase or decrease if the change ≥ ± 15% of glucose uptake relative to the reference condition. These quantization criteria were primarily based on the discussions in the references of the data sources. For instance, in [6] the authors consistently used 2-fold changes to determine differential expression; in [9] zwf was reported up-regulated in pykF knock-out, while the expression ratio of zwf was about 1.2. Ideally quantization should be based on statistical analysis of the control experimental data. Since the control data and statistical analysis were not provided in these sources, we could only use these subjective criteria. The problem of identifying noisy fluctuations as significant changes is less severe as we limited to the subset of genes and reactions highly relevant to the perturbed processes.

### Paths in the joint network

A key concept of this work is to explain perturbation responses with paths in the joint network. In this section we clarify several terms regarding paths and give them mechanistic interpretations. A *metabolic pathway *denotes a series of metabolic reactions with known biochemical functions, e.g., glycolysis or TCA cycle. It is recognized by biochemists and reported in the databases such as EcoCyc. In contrast, a *path *denotes a sequence of consecutive edges in the joint network. It is determined by network topology and may not carry real biological meanings. However, we can give each path a causal, mechanistic interpretation in terms of gene regulation and metabolic reactions. For instance, the red path in Figure 1(a) denotes the gene regulatory alteration via the feedback link between metabolite 1 and regulator 1. The green path in Figure 1(a) denotes the cascade of kinetic shifts along reactions 1–3 by changing metabolite 1. The paths containing only (enzyme, flux), (metabolite, flux) and (flux, metabolite) edges represent the kinetic shifts of reaction fluxes and metabolites due to the changes of metabolites or enzyme levels. We call such paths *metabolic paths*. The paths containing only (regulator, operon) and (operon, gene) edges represent the causal events of transcription regulation. We call these paths *regulatory paths*. We are interested in the causal events which involve both metabolic shifts and gene regulation. A path containing both regulatory and metabolic edges and the (metabolite, regulator) feedback links is called a *mixed path*. A *perturbation response *(or data) denotes a tuple of the perturbation (the change of genes or metabolites), an affected gene or flux, and the direction of the response (up or down regulation, increasing or decreasing flux) with respect to a reference condition. A *valid path *represents a plausible mechanism that can possibly explain a perturbation effect. These paths are obtained by filtering all the paths connecting each perturbation source and response according to node types and network topology. The filtering criteria are described in the Methods. A valid path *explains *a perturbation data if its *predicted response *coincides with the actual response. A valid path *contradicts *with a perturbation data if its predicted response disagrees with the actual response. A valid path is *active *if its underlying mechanism is responsible for the perturbation effect. Concrete definitions of valid paths, predicted responses, explanation and active paths will be given below.

Paths are useful for explaining perturbation responses. A perturbation data contains both direct and indirect effects. The active paths provide consistent hypotheses about the direct and indirect mechanisms for perturbation responses. However, it is easy to create many arbitrarily long paths to fit the perturbation data. To restrict the expressive power of the model we limit the maximum path length and investigate the change of inference results with respect to the path length limit.

### Attributes of the joint network

We assign the following attributes to the joint network: the presence or absence of each edge, the function (sign) of each edge, and the activity of each valid path. Some of these attributes are already known. For example, the sign of an (enzyme, flux) edge is always positive because the enzyme catalyzes the reaction; the signs of many (regulator, operon) edges are reported in EcoCyc. Other attributes are not given thus have to be inferred from data. We are interested in the following unknown attributes: the presence/absence and functions of (metabolite, regulator) edges, and the activities of valid paths. A complete specification of the values of all attributes in the network is called a *model configuration*. It represents a hypothesis about the functions of interactions and causal mechanisms to explain the perturbation data.

### A probabilistic graphical model over network attributes

A perturbation response can be caused by cascades of regulatory changes and metabolic shifts represented by paths in the joint network. To explain a perturbation response with a valid path the predicted response along the path must be consistent with the observed response. The predicted response along a path is determined by the functional direction of the perturbation (increase or decrease), the functions (signs) of (regulator, operon) and (metabolite, regulator) edges, and the causal directions of the reactions (whether the direction of the corresponding flux is identical to or opposite of the "preferred direction" of the reaction). The concrete rules of prediction are described in Methods.

**Figure 2:**
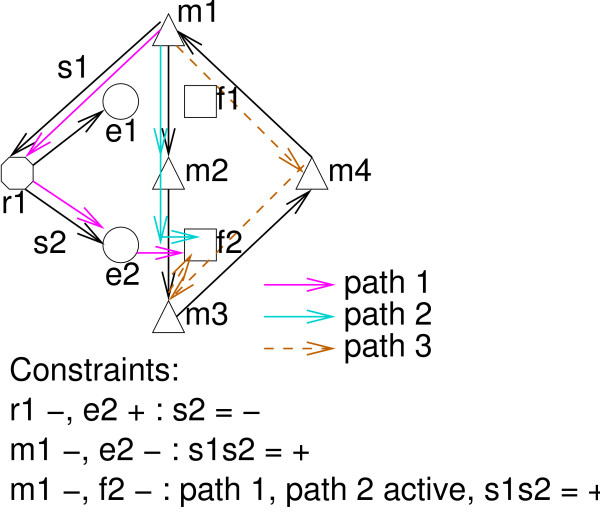
**Constraints on network attributes**. Constraints on network attributes from three perturbation responses. Response 1: deleting regulator *r*_1 _up-regulates enzyme *e*_2_. Constraint: edge sign *s*_2 _= -. Response 2: reducing metabolite *m*_1 _down-regulates enzyme *e*_2_. Constraint: *s*_1_·*s*_2 _= +. Response 3: reducing metabolite *m*_1 _decreases flux *f*_2_. Constraint: path 1 (magenta) and path 2 (cyan) are active, *s*_1_·*s*_2 _= +. Path 3 (brown) is inactive because its predicted response contradicts with the observed response.

The consistency requirement for explaining a perturbation response imposes constraints on the attributes along the path. Figure 2 illustrates these constraints with a toy example. This small network is constrained by three perturbation responses. First, deleting regulator *r*_1 _up-regulates enzyme *e*_2_. The edge sign *e*_2 _is negative in order to explain this response. Second, reducing metabolite *m*_1 _down-regulates *e*_2_. To explain this response the product of *s*_1 _and *s*_2 _is positive. Third, reducing *m*_1 _also reduces flux *f*_2_. *m*_1 _and *f*_2 _are connected by three paths. The predicted responses along paths 1 (magenta) and 2 (cyan) are compatible with the observed response, but the predicted response along path 3 (brown) is the opposite of the observed response. Thus only paths 1 and 2 are active.

The power of integrating constraints is evident even in this toy example. Each local constraint may not suffice to uniquely determine attribute values, but multiple perturbation responses pertaining to overlapped paths may provide sufficient constraints. By combining constraints 1 and 2, we uniquely infer that *s*_1 _and *s*_2 _are both negative. In addition, the responses from one data may reinforce the confidence about path activities for other data. The down-regulation of *e*_2 _in *m*_1 _reduction strongly suggests that path 1 is responsible for explaining the reduction of *f*_2 _in *m*_1 _reduction.

We want to find the attribute values which satisfy all constraints from the perturbation data. This problem is NP-hard and may not be solvable. We relax these hard constraints and express them as a probabilistic graphical model – a factor graph model [25] – over the network attributes. The network attributes are treated as discrete random variables and each local constraint is relaxed as a non-negative real-valued function – a "potential function". The product of all potential functions – the unnormalized joint likelihood function – represents all the constraints from the data. The difficult constraint satisfaction problem is hence relaxed as a tractable graphical model inference problem. The optimal configurations which maximize the joint likelihood function can be calculated using standard inference algorithms such as the max-product algorithm [25]. Details about constructing the factor graph model from local constraints and the inference algorithm are described in Methods.

### Learning feedback links between metabolites and regulators

The feedback links between metabolites and regulators are not reported in the EcoCyc database thus need to be specified. We first applied a simple algorithm to reconstruct these feedback links from perturbation data. It searches among all (metabolite, regulator) pairs and incrementally augments the joint network with the links which maximize the number of explained perturbation responses minus the number of contradicted responses. Intuitively, a link is chosen if it creates "shortcuts" connecting many perturbation data with valid paths. Different sets of links are retrieved at different path length limits, and at a fixed path length limit there can be multiple link sets which have equal explanatory power. We call these link sets degenerate link sets since they are equally good to fit the existing data. Table 2 shows the inferred feedback link sets with the corresponding maximum path length, the explanatory power of the models (# explained responses – # contradicted responses), and their statistical significance using a permutation test. Two interesting properties arise from Table 2. The explanatory power of the model first increases with increasing maximum path length then decreases when the maximum path length exceeds 8. Allowing longer paths connects more perturbation responses but also generates more spurious paths. The model's explanatory power is a balance between these two factors. In addition, the metabolites of the degenerate link sets are close in the metabolic network. For instance, either (glucose, ArcA) or (glucose-6-phosphate, ArcA) appears in many inferred edge sets. Since the existing data do not have sufficient resolution to delineate these links, they tend to explain the same set of perturbation responses. Most optimal link sets in Table 2 comprise two links. The first link connects an upstream metabolite of glycolysis (glucose, glucose-6-phosphate, fructose-6-phosphate) to ArcA, a global repressor of glucose metabolism enzymes [26]. The second link connects a downstream metabolite of glycolysis (pyruvate, acetyl-CoA) to ArcA. These links explain the the expression data of glucose limitation and acetate supply because they connect glucose and acetate to differentially expressed genes. They also create short paths connecting deleted enzymes (pgi, pykA, pykF, zwf) and metabolic fluxes and provide alternative explanation for flux changes.

ArcA and ArcB form a two-component regulatory system for respiratory control [26]. In the deficiency of oxygen, ArcB phosphorylates ArcA, which inhibits the expression of enzymes for aerobic respiration. Apparently ArcA is affected by oxygen but not the metabolites in the central carbon metabolism. However, other links between metabolites and regulators yield inferior explanatory power than those two putative links. For example, a standard mechanism for catabolite repression of enzyme genes is through the CRP-cAMP transcription factor complex [5]. Some genes differentially expressed in glucose limitation – such as fumA, fumC, aceA, aceK, and NADH dehydrogenase members – are not bound by CRP but by ArcA. The link (glucose, CRP) thus cannot explain these expression responses. Previous studies also confirm the effect of acetate and pyruvate on ArcB and ArcA activities [27]. Curiously, acetate and pyruvate are reported to activate ArcA, hence repress the aerobic genes. This prediction contradicts with the expression data of acetate supply, suggesting other regulators are involved or ArcA is affected by conditions accompanied glucose or acetate supply.

To further investigate the properties of inferred models, we focused on a joint network comprising representative links from these two degenerate sets: (glucose, ArcA) and (acetyl-CoA, ArcA). We examined the change of explanatory power of the model with respect to maximum path length and the types of perturbation responses explained by the model. Finally, we investigated the active paths explaining the perturbation data.

The first two rows of Table 3(a) show the net number of explained significant perturbation effects (# explained – # contradicted) and their permutation p-values versus the maximum path length. The explanatory power of the model first increases with the maximum path length then stabilizes when the maximum path length exceeds 6. This property is again a balance between the improved connectivity of models with longer paths and the likely contradictions caused by spurious paths.

**Figure 3:**
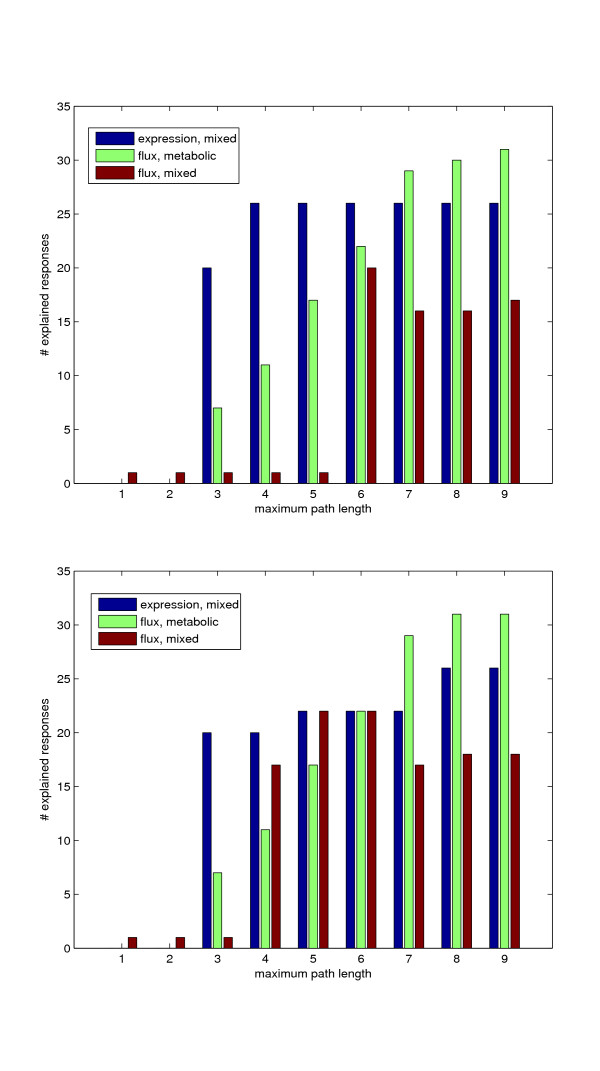
**Types of explained responses (a) inferred links (b) general hypotheses**. The significant perturbation responses explained by each model are categorized into three types: expression changes explained by paths containing both regulatory and metabolic links (dark blue), flux changes explained by pure metabolic paths (green), flux changes explained by paths containing both regulatory and metabolic links (brown). The model of inferred links contains two feedback links (glucose, ArcA), (acetyl-CoA, ArcA).

Figure 3(a) categorizes the significant perturbation effects explained by the models according to two criteria: the type of responses (flux or gene expression) and the type of paths explaining the effects (pure regulatory, pure metabolic, mixed). A central question concerning a joint model of metabolic and regulatory networks is whether the joint model can explain the data considerably better than the individual networks alone. Figure 3(a) and the last two rows of Table 3(a) justify the advantage of the joint model. When the maximum path length is 6, 46 significant responses are explained by mixed paths, whereas only 22 responses are explained by pure metabolic paths. The explanatory power of mixed paths is statistically significant: the permutation p-value of the number of explained responses < 10^-5^. Furthermore, the advantage of mixed paths sustains for the maximum path length from 3 to 9: the permutation p-value ≤ 1.1 × 10^-3 ^for path length 3 and < 10^-5 ^for path length 4–9. All the expression data are explained by mixed paths. Flux data are primarily explained by metabolic paths when the path length < 6, but some of them are explained by mixed paths when the maximum path length ≥ 6. In principle, all the flux responses in the dataset can be explained by pure metabolic paths if the paths are long enough. However, (metabolite, regulator) links establish "shortcuts" connecting sources and responses via regulatory links. These shortcuts bypass the lengthy cascade of metabolic reactions. The results in Figure 3(a) and Table 3(a) are summarized in the supplementary file [see Additional file 13].

To understand the mechanistic interpretations of our models, we further analyzed the active paths responsible for explaining the data from each perturbation experiment. To save space we only present the analysis of 4 perturbation experiments. The active paths explaining each of the 6 perturbation experiments are shown in the supplementary files [see Additional files 1, 2, 3, 4, 5, 6]. All the network graphs are drawn by Cytoscape [28].

**Figure 4:**
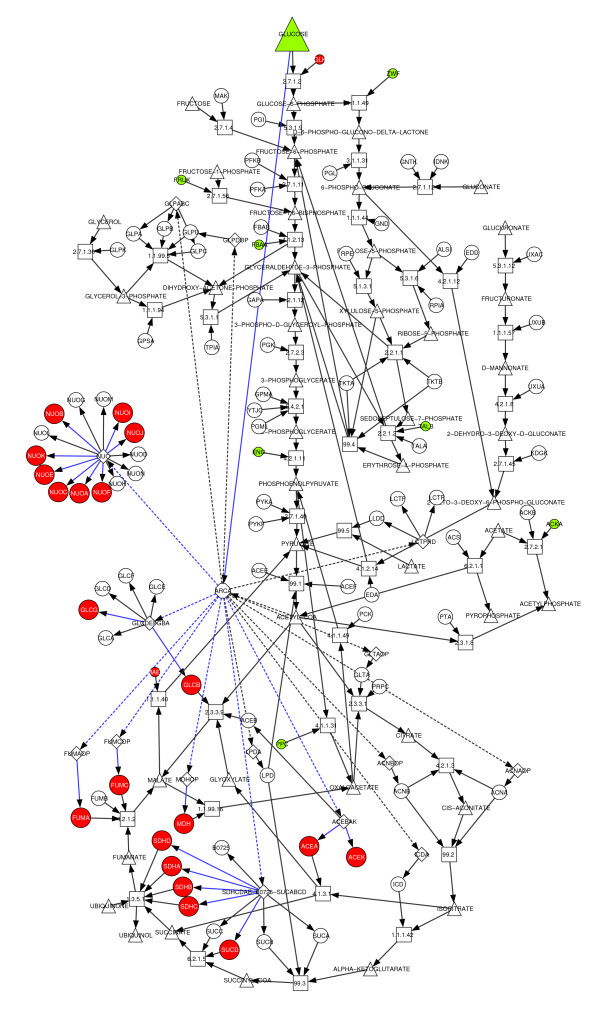
**Active paths explaining glucose limitation, expression data, model of inferred links**. Metabolites (triangles) connect to the fluxes (squares) of the reactions they participate in. Arrows are from the reactants of a reaction to its flux and from the flux of a reaction to its products. The preferred direction of each reaction is specified in EcoCyc. Enzymes (circles) connect to the fluxes they catalyze. Regulators (octagons) connect to operons (diamonds) they regulate, and operons connect to their member genes (circles). Metabolites connect to regulators via the feedback links. Perturbation sources and responses are colored by red (increase) or green (decrease). Enlarged colored nodes denote perturbation sources (glucose in this figure), colored nodes slightly larger than uncolored ones denote the significant responses explained by the model, and small colored nodes denote the unexplained responses. Solid edges are positive (activating), and dash edges are negative (inhibitory). Active paths are marked by blue edges connecting source to responses. For instance, the path **glucose **→ **ArcA **→ **aceBAK (operon) **→ **aceA **explains the up regulation of aceA in glucose limitation.

Figure 4 shows the paths explaining the gene expression responses under glucose limitation relative to amino acid limitation. Glucose is the input of the central carbon metabolism. A few enzymes along glycolysis are down-regulated, and enzymes of the TCA cycle and NADH dehydrogenase (nuoA-nuoK) are up-regulated. Most other genes (white circles in Figure 4) are unmeasured rather than unchanged. The up-regulation of TCA enzymes and NADH dehydrogenase components is explained by mixed paths containing (glucose, ArcA) and from ArcA to corresponding operons. Mechanistically, these paths suggest glucose limitation inhibits ArcA and activates the genes repressed by ArcA. We can thus infer the sign of (glucose, ArcA) is positive.

**Figure 5:**
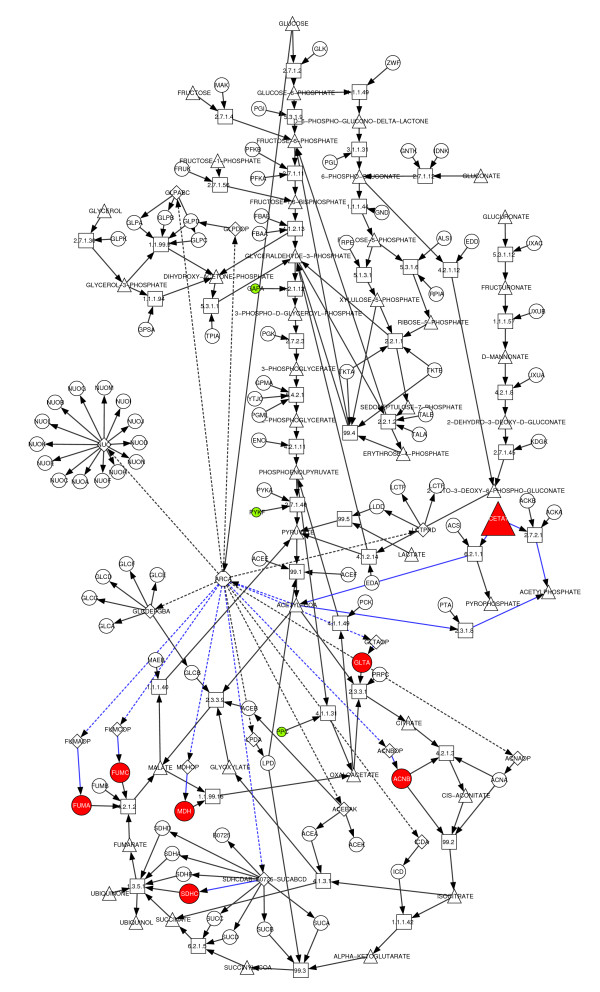
**Active paths explaining acetate supply, expression data, model of inferred links**. Legends are the same as Figure 4.

Figure 5 shows the paths explaining the gene expression responses by supplying cells with acetate as the carbon source. Acetate participates in the central carbon metabolism by converting into acetyl-CoA, the end product of glycolysis and a reactant in the TCA cycle. Most enzymes along the TCA cycle are up-regulated under acetate supply. These responses are explained by paths containing the feedback link (acetyl-CoA, ArcA). Mechanistically, these paths suggest acetate supply increases the level of acetyl-CoA, which inhibits ArcA and activates genes repressed by ArcA. We can also uniquely infer the sign of (acetyl-CoA, ArcA) is negative.

**Figure 6:**
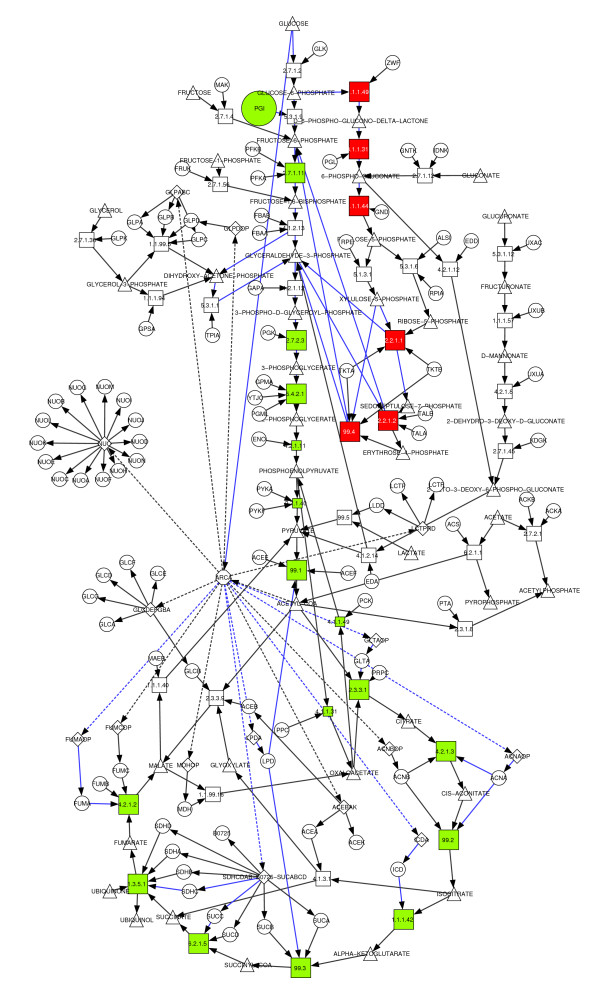
**Active paths explaining pgi knock-out, flux data, model of inferred links**. Legends are the same as Figure 4.

Figure 6 shows the paths explaining the flux responses of deleting pgi, the enzyme catalyzing the second step of glycolysis (EC # 5.3.1.9). Fluxes along glycolysis and the TCA cycle are reduced, and fluxes along pentose phosphorylation are increased. The flux changes along glycolysis and pentose phosphorylation can be explained by pure metabolic paths. Deleting pgi blocks the initial step of glycolysis, which in turn reduces the downstream fluxes of glycolysis and increases the fluxes of pentose phosphorylation which shares the input (glucose-6-phosphate) with glycolysis. The responses of fluxes along the TCA cycle may also be explained by pure but longer metabolic paths. Our model uses shorter, mixed paths containing the feedback link (glucose, ArcA) to explain these interactions. Deleting pgi accumulates the initial metabolites of glycolysis (glucose or glucose-6-phosphate), which enables ArcA, represses enzymes in the TCA cycle, and reduces the fluxes of their catalyzed reactions. The predicted changes along these mixed paths are identical to the predicted changes along pure metabolic paths. Pure metabolic paths are not used by the model because they exceed the path length limit. For example, the metabolic path connecting pgi and flux 4.2.1.2 contains 18 reactions.

**Figure 7:**
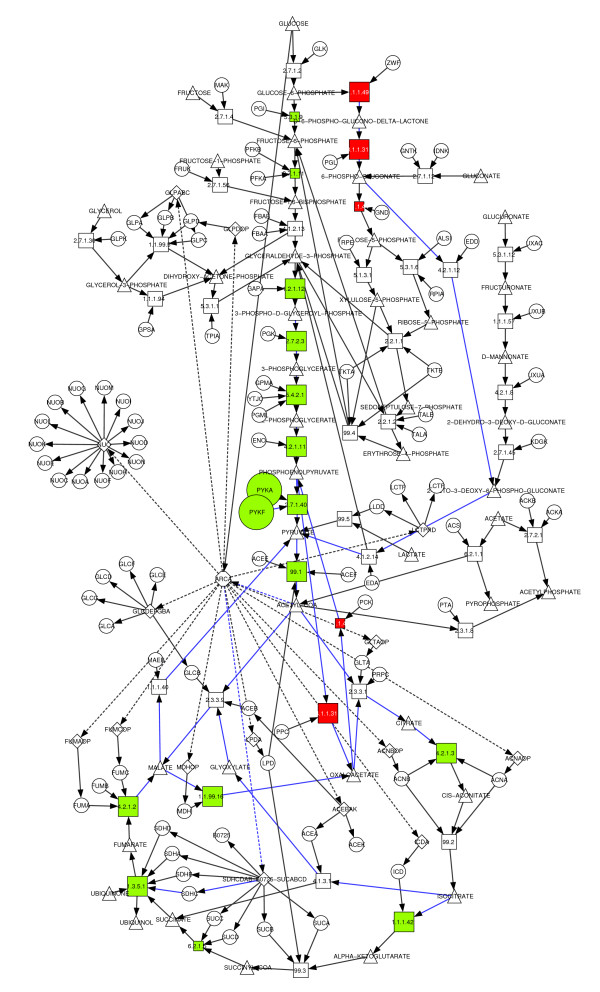
**Active paths explaining pykA/pykF double knock-out, flux data, model of inferred links**. Legends are the same as Figure 4.

Figure 7 shows the paths explaining the flux responses of deleting pykA and pykF, isozymes catalyzing the last step of glycolysis (EC # 2.7.1.40). The effect of blocking this reaction is propagated both upstream and downstream. According to the prediction rules, both upstream and downstream fluxes will decrease. The increase of fluxes along pentose phosphorylation can be explained by paths traversing the common input of glycolysis and pentose phosphorylation (glucose-6-phosphate) or the common output of both pathways (pyruvate). The model uses the latter because its path length is within the upper limit. A few fluxes along the TCA cycle are explained by either (acetyl-CoA, ArcA) link or shorter metabolic paths.

### Encoding general hypotheses about metabolic enzyme regulation

Besides individual links between metabolites and regulators, it is also of interest to know whether enzyme expressions and functions are related by abstract "design rules" or general hypotheses. These rules bypass the incomplete transcription factor binding information and may reveal the general relations between the functions and regulatory architecture of metabolic pathways. Ideally the general hypotheses should be learned from the data as the (metabolite, regulator) links. To do this we need a rigorous definition of hypotheses and a systematic way to search and test these hypotheses. In this preliminary work, we only proposed four simple hypotheses and tested them using the same perturbation data. We consider metabolic pathways with definite physiological functions and preferred directions (e.g., glycolysis, pentose phosphorylation).

1. The input metabolites of a metabolic pathway activate the expression of enzymes catalyzing reactions along the pathway.

2. The end products of a metabolic pathway inhibit the expression of enzymes catalyzing reactions along the pathway.

3. When multiple pathways compete for the same inputs but have different products, the products of one pathway inhibit the enzyme expression along the competing pathway.

4. Glucose represses genes involved in the TCA cycle, electron transfer, and metabolism of other carbon sources.

Hypotheses 1–3 reflect simplified heuristics for efficient resource allocation. To avoid wasting enzymes, a metabolic pathway is turned on only when input metabolites are available, and is turned off when it produces sufficient products or when a competing pathway is active. Hypothesis 4 is based on a well known phenomenon of glucose repression [5]. These simple rules by no means suffice to characterize enzyme gene regulation but are good working hypotheses to test. A general hypothesis is encoded in the model by adding links from metabolites to enzyme genes and designating their functions (edge signs). We encoded these hypotheses in the joint model and applied it to the same perturbation data. The first two rows of Table 3(b) show the net number of explained significant perturbation responses and its statistical significance versus the maximum path length. Figure 3(b) shows the explained significant responses categorized by the types of responses and paths explaining the effects. Similar to Table 3(a), the net number of explained responses first grows with the maximum path length then saturates. The profile of explained effects is also similar to Figure 3(a). Expression data are exclusively explained by mixed paths, flux data are explained only by metabolic paths when the path length is short. As the path length increases more flux data are explained by mixed paths. The net numbers of explained effects are comparable between the two approaches. The results in Figure 3(b) and Table 3(b) are also summarized in the supplementary file [see Additional file 14].

**Figure 8:**
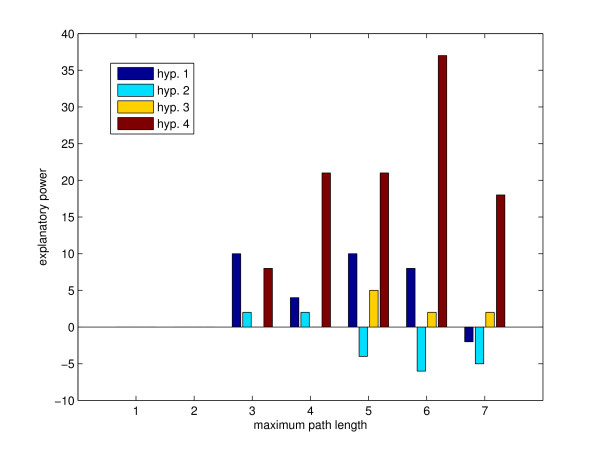
**Contribution of each general hypothesis to the model**. The gain of explaining power by adding each hypothesis into the model. The gain can be negative if adding the hypothesis contradicts with more perturbation responses than explaining them. Hypothesis 1: blue. Hypothesis 2: cyan. Hypothesis 3: yellow. Hypothesis 4: brown.

The explanatory power by adding the general hypotheses to the model is statistically significant across different maximum path length: the p-value < 10^-3 ^for the maximum path length from 5 to 9. We also evaluated the explanatory power contributed by each hypothesis by comparing the model with a null model lacking each hypothesis. Figure 8 shows the gain of the explanatory power contributed by each hypothesis with varying path length. Clearly, hypothesis 4 (glucose repression) has the highest contribution to the explanatory power. Hypothesis 1 (the input of a pathway activates its enzymes) also yields substantial contribution until the maximum path length reaches 7. A negative contribution means the net number of explained responses becomes smaller by incorporating the hypothesis in the model. As the maximum path length increases, more paths are created and the hypothesis is more likely to be violated along some of these paths. Hypothesis 3 (the product of a pathway inhibits the enzymes of the competing pathway) has a small yet non-negative contribution for the maximum path length ≥ 5. In contrast, hypothesis 2 (the output of a pathway inhibits its enzymes) has small positive contribution along short paths but has negative contribution as the maximum path length exceeds 5. Therefore, adding hypothesis 2 to the model does not improve (or even degrades) the explanatory power of the model. We also draw the active paths in the general hypotheses models explaining each perturbation experiments. To save space we put these figures in the supplementary files [see Additional files 7, 8, 9, 10, 11, 12].

### Validating inferred models

We performed two computational tests to validate the predictive power and the consistency of the model learning algorithm. First we performed three cross validation tests on the perturbation data to check whether the model could accurately predict the perturbation responses. Second we built hypothetical models of metabolic-regulatory coupling, simulated perturbation response data according to these models, and verified whether the learning algorithm could recover the hypothetical models.

**Figure 9:**
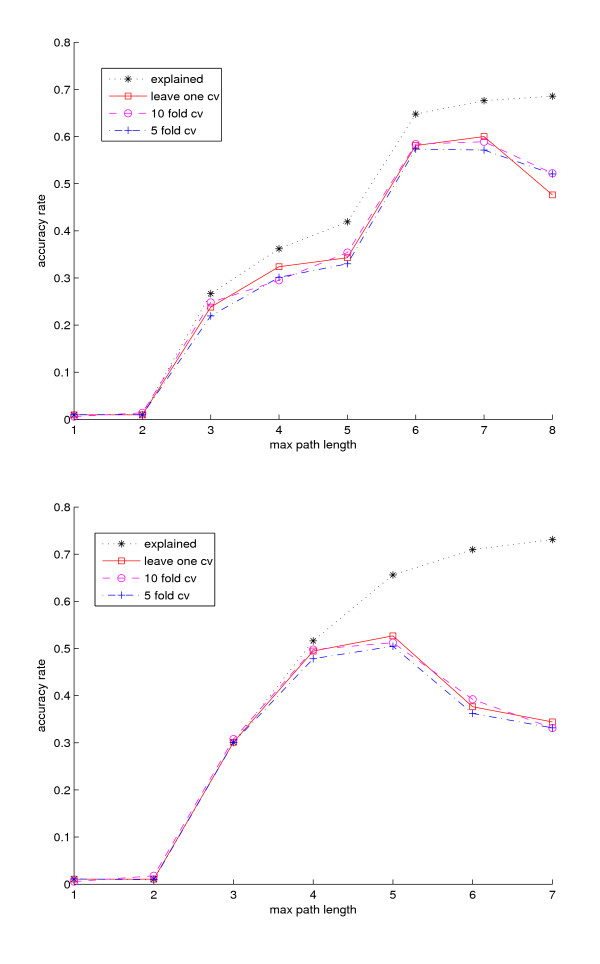
**Cross validation accuracy of models with (a) inferred links and (b) general hypotheses**. Black stars are the accuracy rates using all perturbation data in the training set. Red squares are the accuracy rates of leave-one-out cross validation. Magenta circles and blue crosses are the accuracy rates of 10-fold and 5-fold cross validation tests.

Figure 9 demonstrates the accuracy rates of cross-validation tests by varying the maximum path length in the model. We split the significant perturbation responses into training and test sets, inferred the model configurations from the training data, and predicted the responses in the test data. To reduce the influence from the test data we used three methods to generate test sets. Leave-one cross validation treats each (perturbation, response) tuple as a test set and repeats the procedure for each tuple. 10-fold cross validation randomly chooses one tenth of the tuples as a test set and repeats the procedure for 100 trials, and 5-fold cross validation chooses one fifth of the tuples instead. The accuracy rates of 10-fold and 5-fold cross validation in Figure 9 are the averages over 100 trials. The standard deviations are smaller than 0.16 for 10-fold cross validation and smaller than 0.1 for 5-fold cross validation.

Figure 9(a) shows the results of models with two inferred (metabolite, regulator) links (glucose, ArcA) and (acetyl-CoA, ArcA), and Figure 9(b) shows the results of models encoded with the general hypotheses of metabolic enzyme control. Black dotted lines are the accuracy rates using the entire dataset (1 – training error rate). They are the upper bounds for the test accuracy rates. For the models with the two representative links, the test accuracy rate is close to its upper bound when the maximum path length ≤ 7.

The test accuracy reaches 60% when the maximum path length is 7, and drops significantly to about 50% when the maximum path length is 8. This suggests overfitting occurs as the maximum path length exceeds 7 due to spurious paths. Figure 9(b) shows the results of models derived from the general hypotheses. It demonstrates a similar pattern as Figure 9(a): the accuracy rate is close to their upper bounds when the maximum path length ≤ 4, reaches the maximum around 50% when the maximum path length is 5, and drops significantly as the maximum path length exceeds 5. The inferior test accuracy rates are due to the larger number of valid paths created by the general hypotheses. Moreover, the test accuracy rates generated by three different cross validation tests are very close, indicating they are robust against the choice of test sets.

**Figure 10:**
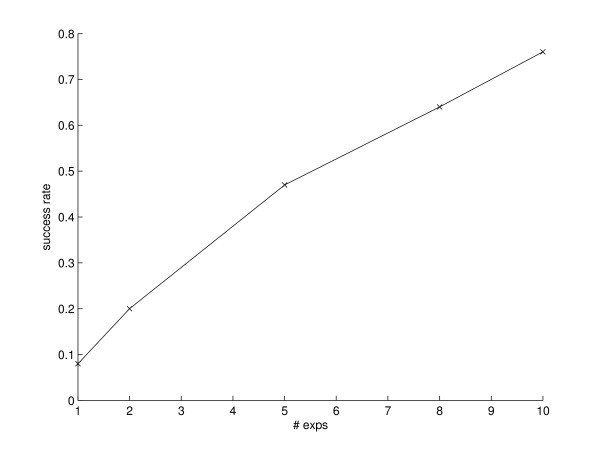
**Success rate of retrieving missing links from simulated data**. In each trial, two random (metabolite, regulator) links are added to the joint network, and their functions are randomly assigned. The maximum path length is 6. The feedback links are counted as accurately learned if they are among the degenerate link sets generated by the algorithm. The success rate is the fraction of random trials which accurately learn the feedback links from simulated data. The success rate increases as the number of perturbation experiments increases.

Besides cross validation, we also verified the consistency of the model learning algorithm by checking whether the algorithm could accurately learn the underlying model if the model hypotheses were correct and sufficient data were given. We generated a hypothetical model by adding two random (metabolite, regulator) links and assigning random signs to these edges. Hypothetical perturbation experiments were selected by randomly deleting/overexpressing genes or increasing/decreasing metabolite levels in the joint network. Expression and flux responses of each perturbation were simulated according to the hypothetical model, and significant responses were incorporated in the hypothetical perturbation data. We then inferred the missing links and their signs from these data and compared the inferred links with the hypothetical model. This procedure was repeated for 200 random hypothetical models. As shown in Figure 10, the fraction of random models accurately learned by the learning algorithm increases with the number of experiments. When 5 experiments were provided, about half (47%) of the random models were accurately learned by the algorithm. When 10 experiments were provided, over three quarters (76%) of the random models were accurately learned. This "learning curve" suggests that our method can in principle learn the missing links (and their functions) if sufficient data are provided.

## Discussion

We want to achieve four goals from the inferred models: 1) evaluate the advantage of an integrated model over separate systems, 2) identify the feedback links between metabolites and regulators and their functions, 3) computationally test the general hypotheses pertaining to the feedback control of enzyme expressions, 4) identify the active paths responsible for perturbation effects. In this section we discuss whether these goals are fulfilled from the results.

The advantage of an integrated model is obvious. As shown in Table 3, a joint model of regulatory and metabolic networks explains perturbation data significantly better than the two separate networks. This advantage arises from the improved connection from perturbation sources to responses in the joint network. Specifically, it is essential to establish feedback links from metabolites to transcription factors or genes in order to explain the differential expression of genes under different metabolic conditions. Other types of perturbation data may also be explained by short paths containing both regulatory and metabolic links. Table 2 indicates multiple sets of feedback links yield the same explanatory power. Therefore, we cannot uniquely identify the feedback links. This is expected since our perturbation data do not have sufficient resolution to discriminate between multiple link sets. Nevertheless, we can approximately map the positions of the metabolites involved in the metabolic network: one link connects an upstream metabolite of glycolysis to ArcA, and the other link connects a downstream metabolite of glycolysis to ArcA. These degenerate link sets can guide us to narrow down the true feedback links by further experiments.

The two inferred links (glucose, ArcA) and (acetyl-CoA, ArcA) are not directly supported from previous studies. The main objection is that ArcA is regulated by oxygen but not metabolites in the central carbon metabolism [26]. We argue the validity of these links from both modeling and biological perspectives. Two criteria of justifying a model are its simplicity and predictive power. These two links are the most "economical" augmentation to explain the data because many genes differentially expressed in glucose limitation and acetate supply are bound by ArcA. More links are required if we consider the feedback control through other transcription factors. For instance, some differentially expressed genes in glucose limitation are not bound by CRP, a standard regulator for glucose repression. Furthermore, cross validation tests indicate the models can accurately predict the perturbation responses. Biologically, the two links may embody indirect mechanisms. Aerobic respiration is elevated by glucose limitation or acetate supply, whereas ArcA is a main repressor for aerobic respiration enzymes. The influence of glucose and acetate on aerobic respiration hence may indirectly modulates ArcA and affects these enzymes.

As shown in Table 3(b), a model encoded with four simple hypotheses explains the data significantly better than the model without these hypotheses. However, we found the explanatory power contributed by each hypothesis was quite different. While the hypothesis of glucose repression (hypothesis 4) accounts for a large number of perturbation responses, hypotheses 1 and 3 have only moderate and weak explanatory power. Moreover, hypothesis 2 apparently yields contradictions as the maximum path length becomes longer. The results imply some of these hypotheses are not valid and more complex "design rules" exist. For the extremely robust, tightly regulated system of central carbon metabolism, there are probably more sophisticated design rules governing gene regulation and metabolic reactions. Therefore, it would be desirable to apply the tests to the data covering a wide range of metabolic pathways.

Finally we want to verify the inferred active paths explaining the perturbation responses. Pure metabolic paths connecting metabolites or enzymes to fluxes merely restate the kinetic shifts of metabolic reactions. Mixed paths, however, are difficult to verify directly. We again justify these paths from both modeling and biological perspectives. From a modeling perspective, we want to restrict the path length to limit model complexity and reduce overfitting. Therefore, we tend to use "shortcuts" via the feedback links to explain the perturbation responses. On the other hand, the active paths explaining different types of perturbation data are highly overlapped. For instance, in Figures 4 and 6 we use paths containing (glucose, ArcA) and the links from ArcA to TCA enzymes to explain both the expression data in glucose limitation and flux data in pgi knock-out. Similarly, in Figures 5 and 7 we use paths containing (acetyl-CoA, ArcA) and the links from ArcA to TCA enzymes to explain both the expression data in acetate supply and flux data in pykA/pykF knock-outs. Using the same set of mechanistic hypotheses to explain different data is desirable from a modeling perspective since it suggests the model is not tuned to a specific data.

Biologically, the inferred active paths provide insight about the underlying mechanisms. One interesting example is the paths explaining the reduction of TCA fluxes under pgi knock-out (Figure 6). We used the mixed paths containing (glucose, ArcA) and (ArcA, TCA enzymes) to explain the flux changes because they are much shorter than the pure metabolic paths. The longer metabolic paths are indeed responsible for these flux changes since a long cascade of kinetic shifts can occur rapidly. However, we should not exclude the influence of enzyme gene regulation in flux changes. As shown in Figures 4, 5, 6, 7, altering metabolic conditions changes both metabolic fluxes and enzyme levels. Thus flux changes are likely to be the composite effects of both kinetic readjustment and gene regulation. Intriguingly, our model indicates the directions of these two effects are consistent. For example, blocking the second step of glycolysis (pgi knock-out, Figure 6) has the opposite effect of reducing the input metabolite of glycolysis (glucose limitation, Figure 4). The metabolic fluxes along the TCA cycle are decreased in pgi knock-out and the enzyme expressions along the TCA cycle are up-regulated in glucose limitation. Similarly, blocking the second last step of glycolysis (pykA/pykF knock-out, Figure 7) has the opposite effect of increasing the output metabolite of glycolysis (acetate supply, Figure 5). The metabolic fluxes along the TCA cycle are decreased in pykA/pykF knock-out and the enzyme expressions along the TCA cycle are up-regulated in acetate supply. The agreement between enzyme expression changes and flux readjustment suggests gene regulation exerts another layer of control enhancing the kinetic shifts of the metabolic network. This design is sensible from an evolutionary perspective since gene regulation is likely evolved to improve the efficiency of an existing system of biochemical reactions.

The main contribution of this work is model the coupled processes of gene regulation and metabolism with a simple, mechanistic abstraction – paths in the joint network. The analysis on the data of the central carbon metabolism provides biological insight about the underlying system. As a proof-of-concept demonstration we do not expect to have novel discoveries from this very well-studied system. We plan to apply the model to less well-known systems such as the metabolism of human, other bacteria or archea. We are also aware of various limitations of the model: the discretized model attributes and data, ignorance of the dynamics of gene regulation and biochemical reactions, simplification of the combinatorial regulation of multiple transcription factors, and so on. We plan to overcome these limitations in the extended version of the model.

## Conclusion

We propose a unified modeling framework of integrating the information of gene regulation and metabolic reactions. We hypothesize the perturbation effects are propagated along paths in the joint network and build a factor graph model specifying the constraints of using these paths to explain the perturbation data. The learning algorithm identifies the feedback links between metabolites and transcription factors, their functions and the active paths explaining the perturbation data. We also explicitly test four general hypotheses relating the functions and regulation of enzymes. By applying the model to the expression and flux data of the central carbon metabolism, we suspect gene regulation provides another layer of control to enhance the rebalance of reactions toward the new steady states. We also show the four simple hypotheses have different explanatory power to fit the data and suggest more sophisticated design rules may govern the regulation of the central carbon metabolism.

## Methods

### Valid paths in the joint network

A path in the joint network represents a series of mechanisms of gene regulation or metabolic reactions. Not all paths can possibly explain the perturbation data. For example, a path is irrelevant if it does not connect the perturbation source and the effect. We define a valid path as a plausible mechanism that can possibly explain a perturbation effect. Specifically, we require a valid path satisfies the following conditions:

1. The source of the path is the perturbed gene/metabolite and destination is the affected gene/flux.

2. The path does not contain repeated nodes.

3. If multiple paths connect a (perturbation, response) pair, and one path subsumes the other, then discard the longer path.

4. The path does not contain repeated edges or (metabolite, flux) edges in the opposite directions.

5. The path does not contain the consecutive edges (metabolite1, flux), (flux, metabolite2) where metabolite1 and metabolite2 are at the same side of a reaction.

6. If multiple paths connect a (perturbation, response) pair and two paths traverse the same reaction along opposite directions, then only keep the paths in one reaction direction.

7. Avoid using the paths whose intermediate nodes are perturbed and the responses of downstream nodes are insignificant.

8. The path length is not longer than an upper limit.

Condition 1 is obvious. Conditions 2 and 3 are parsimonious. Conditions 4, 5, 6 guarantee the perturbation effects are not propagated along both directions of the same reaction. Condition 7 follows the assumption that each gene or flux along a path responds to the perturbation. Condition 8 restricts the explanatory power of the model. This restriction is necessary since any (perturbation, response) tuple is likely to be connected by arbitrarily long paths in the joint network.

### Predicting a perturbation response along a valid path

**Figure 11:**
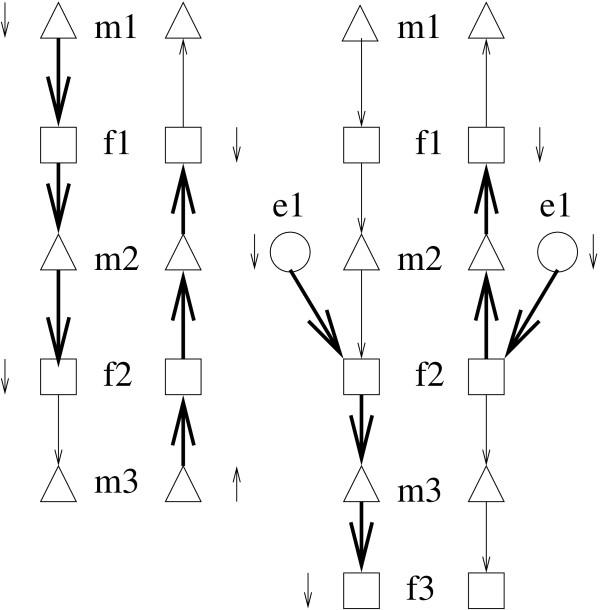
**Predictions along metabolic paths**. Bold lines indicate paths connecting the perturbation source to the response. path 1: metabolite *m*_1 _is limited, flux *f*_2 _is reduced. path 2: metabolite *m*_3 _is supplied, flux *f*_1 _is reduced. path 3: enzyme *e*_1 _is deleted, flux *f*_3 _is reduced. path 4: enzyme *e*_1 _is deleted, flux *f*_1 _is reduced.

We can predict the perturbation response along a valid path if the direction of perturbation and the edge signs along the path are given. Here perturbations and responses are quantized into three levels {-1, 0, +1} (decrease, no change, increase). We decompose a path into regulatory subpaths (consecutive (regulator, operon), (operon, gene) edges), metabolic subpaths (consecutive (enzyme, flux), (flux, metabolite), (metabolite, flux) edges), and the coupling parts ((metabolite, regulator) edges). The aggregate effect along the regulatory subpaths and the coupling parts is the product of the perturbation direction and the signs of (regulator, operon), (operon, gene), and (metabolite, regulator) edges. The aggregate effect along a metabolic subpath is determined by the functional direction (increase or decrease) of the perturbation and the causal directions (whether the direction of the corresponding flux is identical to or opposite of the "preferred direction" of the reaction) of the first and last reactions along the subpath. Figure 11 illustrates four cases of the aggregate effect along a metabolic subpath. Decreasing a metabolite *m *will decrease the fluxes of the reactions which take *m *as an input. The effect may traverse along (path 1 in Figure 11) or against (path 2 in Figure 11) the preferred directions of reactions. Decreasing an enzyme *e *reduces the outputs and accumulates the inputs of its catalyzing reaction. The effects of reducing the outputs (path 3 in Figure 11) and increasing the inputs (path 4 in Figure 11) are propagated downstream and upstream respectively. In addition, because a metabolic flux is defined by a reference direction (the preferred direction of the reaction), one has to invert the predicted response when the direction of the last reaction along the path is the opposite of its reference direction. By combining these rules we can express the prediction along a metabolic subpath in the following form. Denote *p *the functional direction of the perturbation, *f *the response of the terminal flux (relative to the preferred direction), *d*_1 _the (causal) direction of the first reaction along the path (relative to the preferred direction), and *d*_*n *_the causal direction of the last reaction along the path. The predicted response is

f={−p⋅dnif (perturb enzyme)∧(d1=−1),p⋅dnif (perturb metabolite)∨(d1=1)     (1)
 MathType@MTEF@5@5@+=feaafiart1ev1aaatCvAUfKttLearuWrP9MDH5MBPbIqV92AaeXatLxBI9gBaebbnrfifHhDYfgasaacH8akY=wiFfYdH8Gipec8Eeeu0xXdbba9frFj0=OqFfea0dXdd9vqai=hGuQ8kuc9pgc9s8qqaq=dirpe0xb9q8qiLsFr0=vr0=vr0dc8meaabaqaciaacaGaaeqabaqabeGadaaakeaacqWGMbGzcqGH9aqpdaGabaqaauaabaqaciaaaeaacqGHsislcqWGWbaCcqGHflY1cqWGKbazdaWgaaWcbaGaemOBa4gabeaaaOqaaiabbMgaPjabbAgaMjabbccaGiabcIcaOiabbchaWjabbwgaLjabbkhaYjabbsha0jabbwha1jabbkhaYjabbkgaIjabbccaGiabbwgaLjabb6gaUjabbQha6jabbMha5jabb2gaTjabbwgaLjabcMcaPiabgEIizlabcIcaOiabdsgaKnaaBaaaleaacqaIXaqmaeqaaOGaeyypa0JaeyOeI0IaeGymaeJaeiykaKIaeiilaWcabaGaemiCaaNaeyyXICTaemizaq2aaSbaaSqaaiabd6gaUbqabaaakeaacqqGPbqAcqqGMbGzcqqGGaaicqGGOaakcqqGWbaCcqqGLbqzcqqGYbGCcqqG0baDcqqG1bqDcqqGYbGCcqqGIbGycqqGGaaicqqGTbqBcqqGLbqzcqqG0baDcqqGHbqycqqGIbGycqqGVbWBcqqGSbaBcqqGPbqAcqqG0baDcqqGLbqzcqGGPaqkcqGHOiI2cqGGOaakcqWGKbazdaWgaaWcbaGaeGymaedabeaakiabg2da9iabigdaXiabcMcaPaaacaWLjaGaaCzcaiabcIcaOiabigdaXiabcMcaPaGaay5Eaaaaaa@88E0@

The predicted response along a mixed path is determined by sequentially predicting the response of each regulatory, metabolic and coupling subpath and taking the predicted response of one subpath as the perturbation of the next subpath.

### A factor graph model over network attributes

To explain a perturbation data we require at least one path connecting each perturbation source and the response is active, and predicted responses along active paths are consistent with the actual response. In addition, the regulatory activities along the paths should respect the operon structure. These requirements impose various constraints on the network attributes. We relax these hard constraints and express them as a probabilistic graphical model – a factor graph model [25] – over the network attributes. Similar modeling techniques have been applied to the physical network [29], protein-protein interactions [30], and gene regulation [31]. We model each attribute as a discrete random variable and introduce the following notation: *X *as the collection of all edge presence/absence attributes which take values in {0, 1}, *S *as the collection of all edge sign attributes which take values in {-1, +1}, and *A *as the collection of all path activity attributes which take values in {0, 1}. The (unnormalized) joint likelihood function of a factor graph is the product of "potential functions", where each potential function represents a relaxed constraint derived from data and model assumptions.

We build three types of potential functions in the model. To explain that a gene or a metabolic flux is altered in a perturbation, we require there exists valid paths to propagate the perturbation effects and the predicted effects along these paths are consistent with the observed response. Specifically, a (perturbation, response) pair is explained by the model if the following conditions hold:

1. Each perturbed source is connected to the response via at least one valid path.

2. At least one valid path connecting each source to the response is active.

3. All edges along active paths are present.

4. The prediction of the perturbation effect along each active path is consistent with the observed response.

Condition 1 sustains by identifying all valid paths connecting each (perturbation, response) pair. The predicted response along a path is determined by the perturbation and edge signs along the path. Furthermore, the constraints of edge presence and edge signs are relaxed if the path is not active. For a path *π*, these conditions are expressed as the following potential function:

φπ(Xπ,Sπ,aπ;p,d)={1−εif (aπ=1)∧(∀xi∈Xπ,xi=1)∧(pred(p,Sπ)=d)1−εif (aπ=0)εotherwise.     (2)
 MathType@MTEF@5@5@+=feaafiart1ev1aaatCvAUfKttLearuWrP9MDH5MBPbIqV92AaeXatLxBI9gBaebbnrfifHhDYfgasaacH8akY=wiFfYdH8Gipec8Eeeu0xXdbba9frFj0=OqFfea0dXdd9vqai=hGuQ8kuc9pgc9s8qqaq=dirpe0xb9q8qiLsFr0=vr0=vr0dc8meaabaqaciaacaGaaeqabaqabeGadaaakeaaiiGacqWFgpGzdaWgaaWcbaGae8hWdahabeaakmaabmaabaGaemiwaG1aaSbaaSqaaiab=b8aWbqabaGccqGGSaalcqWGtbWudaWgaaWcbaGae8hWdahabeaakiabcYcaSiabdggaHnaaBaaaleaacqWFapaCaeqaaOGaei4oaSJaemiCaaNaeiilaWIaemizaqgacaGLOaGaayzkaaGaeyypa0ZaaiqaaeaafaqaaeWacaaabaGaeGymaeJaeyOeI0Iae8xTdugabaGaeeyAaKMaeeOzayMaeeiiaaIaeiikaGIaemyyae2aaSbaaSqaaiab=b8aWbqabaGccqGH9aqpcqaIXaqmcqGGPaqkcqGHNis2cqGGOaakcqGHaiIicqWG4baEdaWgaaWcbaGaemyAaKgabeaakiabgIGiolabdIfaynaaBaaaleaacqWFapaCaeqaaOGaeiilaWIaemiEaG3aaSbaaSqaaiabdMgaPbqabaGccqGH9aqpcqaIXaqmcqGGPaqkcqGHNis2cqGGOaakcqWGWbaCcqWGYbGCcqWGLbqzcqWGKbazcqGGOaakcqWGWbaCcqGGSaalcqWGtbWudaWgaaWcbaGae8hWdahabeaakiabcMcaPiabg2da9iabdsgaKjabcMcaPaqaaiabigdaXiabgkHiTiab=v7aLbqaaiabbMgaPjabbAgaMjabbccaGiabcIcaOiabdggaHnaaBaaaleaacqWFapaCaeqaaOGaeyypa0JaeGimaaJaeiykaKcabaGae8xTdugabaGaee4Ba8MaeeiDaqNaeeiAaGMaeeyzauMaeeOCaiNaee4DaCNaeeyAaKMaee4CamNaeeyzauMaeeOla4caaaGaay5EaaGaaCzcaiaaxMaacqGGOaakcqaIYaGmcqGGPaqkaaa@967C@

where *X*_*π *_and *S*_*π *_denote the collection of edge presence and edge signs along *π*, *a*_*π *_the activity of path *π*, *p *and *d *directions of perturbation and response given by the data, and *pred*(*p*, *S*_*π*_) denotes the aforementioned prediction of perturbation *p *according to edge signs *S*_*π*_. It returns 1 – *ε *when *π *is active, all edges are present, and the predicted response along *π *is consistent with the observed response, or when *π *is not active. It penalizes the attribute values where *π *is active but cannot explain the perturbation effect. When there are multiple perturbation sources (e.g., double knock-outs) or multiple paths connecting a (perturbation, response) pair, condition 2 requires each perturbation source has at at least one active path connecting to the response. This requirement translates into the following potential functions pertaining to path activities. Denote (*p*, *d*) a tuple of perturbation *p *and response *d*, *A*_1_,..., *A*_*k *_as the activities of paths connecting each source 1,..., *k *to the response, and *A*_(*p*,*d*) _= ∪_*i*_*A*_*i*_. The potential function of path activities is

φ(p,d)(A(p,d))={1−εif (∃a1∈A1,a1=1)∧⋯∧(∃ak∈Ak,ak=1),εotherwise.     (3)
 MathType@MTEF@5@5@+=feaafiart1ev1aaatCvAUfKttLearuWrP9MDH5MBPbIqV92AaeXatLxBI9gBaebbnrfifHhDYfgasaacH8akY=wiFfYdH8Gipec8Eeeu0xXdbba9frFj0=OqFfea0dXdd9vqai=hGuQ8kuc9pgc9s8qqaq=dirpe0xb9q8qiLsFr0=vr0=vr0dc8meaabaqaciaacaGaaeqabaqabeGadaaakeaaiiGacqWFgpGzdaWgaaWcbaWaaeWaaeaacqWGWbaCcqGGSaalcqWGKbazaiaawIcacaGLPaaaaeqaaOGaeiikaGIaemyqae0aaSbaaSqaamaabmaabaGaemiCaaNaeiilaWIaemizaqgacaGLOaGaayzkaaaabeaakiabcMcaPiabg2da9maaceaabaqbaeaabiGaaaqaaiabigdaXiabgkHiTiab=v7aLbqaaiabbMgaPjabbAgaMjabbccaGmaabmaabaGaey4aIqIaemyyae2aaSbaaSqaaiabigdaXaqabaGccqGHiiIZcqWGbbqqdaWgaaWcbaGaeGymaedabeaakiabcYcaSiabdggaHnaaBaaaleaacqaIXaqmaeqaaOGaeyypa0JaeGymaedacaGLOaGaayzkaaGaey4jIKTaeS47IWKaey4jIKTaeiikaGIaey4aIqIaemyyae2aaSbaaSqaaiabdUgaRbqabaGccqGHiiIZcqWGbbqqdaWgaaWcbaGaem4AaSgabeaakiabcYcaSiabdggaHnaaBaaaleaacqWGRbWAaeqaaOGaeyypa0JaeGymaeJaeiykaKIaeiilaWcabaGae8xTdugabaGaee4Ba8MaeeiDaqNaeeiAaGMaeeyzauMaeeOCaiNaee4DaCNaeeyAaKMaee4CamNaeeyzauMaeeOla4caaaGaay5EaaGaaCzcaiaaxMaacqGGOaakcqaIZaWmcqGGPaqkaaa@7B14@

The joint constraint for explaining a perturbation data (*p*, *d*) is the product of *φ*_(*p*, *d*) _(*A*_(*p*, *d*)_) and the *φ*_*π *_(*X*_*π*_, *S*_*π*_, *a*_*π*_; *p*, *d*) of each path *π *in the set of valid paths ∏_(*p*, *d*) _connecting (*p*, *d*):

φ(p,d)(X,S,A)=φ(p,d)(A(p,d))⋅∏π∈Π(p,d)φπ(Xπ,Sπ,aπ; p,d).     (4)
 MathType@MTEF@5@5@+=feaafiart1ev1aaatCvAUfKttLearuWrP9MDH5MBPbIqV92AaeXatLxBI9gBaebbnrfifHhDYfgasaacH8akY=wiFfYdH8Gipec8Eeeu0xXdbba9frFj0=OqFfea0dXdd9vqai=hGuQ8kuc9pgc9s8qqaq=dirpe0xb9q8qiLsFr0=vr0=vr0dc8meaabaqaciaacaGaaeqabaqabeGadaaakeaaiiGacqWFgpGzdaWgaaWcbaWaaeWaaeaacqWGWbaCcqGGSaalcqWGKbazaiaawIcacaGLPaaaaeqaaOWaaeWaaeaacqWGybawcqGGSaalcqWGtbWucqGGSaalcqWGbbqqaiaawIcacaGLPaaacqGH9aqpcqWFgpGzdaWgaaWcbaWaaeWaaeaacqWGWbaCcqGGSaalcqWGKbazaiaawIcacaGLPaaaaeqaaOGaeiikaGIaemyqae0aaSbaaSqaamaabmaabaGaemiCaaNaeiilaWIaemizaqgacaGLOaGaayzkaaaabeaakiabcMcaPiabgwSixpaarafabaGae8NXdy2aaSbaaSqaaiab=b8aWbqabaGccqGGOaakcqWGybawdaWgaaWcbaGae8hWdahabeaakiabcYcaSiabdofatnaaBaaaleaacqWFapaCaeqaaOGaeiilaWIaemyyae2aaSbaaSqaaiab=b8aWbqabaGccqGG7aWoaSqaaiab=b8aWjabgIGiolabfc6aqnaaBaaameaacqGGOaakcqWGWbaCcqGGSaalcqWGKbazcqGGPaqkaeqaaaWcbeqdcqGHpis1aOGaeeiiaaIaemiCaaNaeiilaWIaemizaqMaeiykaKIaeiOla4IaaCzcaiaaxMaacqGGOaakcqaI0aancqGGPaqkaaa@740E@

*φ*_(*p*, *d*) _(*X*, *S*, *A*) penalizes the attribute values where predicted responses along some active paths contradict with the actual response or no path from some source to the response is active. It therefore encodes conditions 1–4 for explaining a perturbation data.

In addition to the constraints for explaininng perturbation responses, we also require the paths connecting the same perturbation and containing the same operon are either all active or all inactive. This constraint translates into the following potential function. Denote *A*_*op *_the collection of path activities with this property.

φop(Aop)={1−εif (∀a∈Aop,a=1)∨(∀a∈Aop,a=0),εotherwise.     (5)
 MathType@MTEF@5@5@+=feaafiart1ev1aaatCvAUfKttLearuWrP9MDH5MBPbIqV92AaeXatLxBI9gBaebbnrfifHhDYfgasaacH8akY=wiFfYdH8Gipec8Eeeu0xXdbba9frFj0=OqFfea0dXdd9vqai=hGuQ8kuc9pgc9s8qqaq=dirpe0xb9q8qiLsFr0=vr0=vr0dc8meaabaqaciaacaGaaeqabaqabeGadaaakeaaiiGacqWFgpGzdaWgaaWcbaGaem4Ba8MaemiCaahabeaakiabcIcaOiabdgeabnaaBaaaleaacqWGVbWBcqWGWbaCaeqaaOGaeiykaKIaeyypa0ZaaiqaaeaafaqaaeGacaaabaGaeGymaeJaeyOeI0Iae8xTdugabaGaeeyAaKMaeeOzayMaeeiiaaIaeiikaGIaeyiaIiIaemyyaeMaeyicI4Saemyqae0aaSbaaSqaaiabd+gaVjabdchaWbqabaGccqGGSaalcqWGHbqycqGH9aqpcqaIXaqmcqGGPaqkcqGHOiI2cqGGOaakcqGHaiIicqWGHbqycqGHiiIZcqWGbbqqdaWgaaWcbaGaem4Ba8MaemiCaahabeaakiabcYcaSiabdggaHjabg2da9iabicdaWiabcMcaPiabcYcaSaqaaiab=v7aLbqaaiabb+gaVjabbsha0jabbIgaOjabbwgaLjabbkhaYjabbEha3jabbMgaPjabbohaZjabbwgaLjabb6caUaaaaiaawUhaaiaaxMaacaWLjaGaeiikaGIaeGynauJaeiykaKcaaa@70D0@

The joint likelihood function is the product of equation 2 for each path, equation 3 for each perturbation response, and equation 5 for each set of path activities containing the same operon:

L(X,S,A;D)=∏(p,d),A(p,d)~(p,d)φ(p,d)(A(p,d))⋅∏π∈Π(p,d)φπ(Xπ,Sπ,aπ;p,d)⋅∏Aopφop(Aop).     (6)
 MathType@MTEF@5@5@+=feaafiart1ev1aaatCvAUfKttLearuWrP9MDH5MBPbIqV92AaeXatLxBI9gBaebbnrfifHhDYfgasaacH8akY=wiFfYdH8Gipec8Eeeu0xXdbba9frFj0=OqFfea0dXdd9vqai=hGuQ8kuc9pgc9s8qqaq=dirpe0xb9q8qiLsFr0=vr0=vr0dc8meaabaqaciaacaGaaeqabaqabeGadaaakeaacqWGmbatdaqadaqaaiabdIfayjabcYcaSiabdofatjabcYcaSiabdgeabjabcUda7iabdseaebGaayjkaiaawMcaaiabg2da9maarafabaacciGae8NXdy2aaSbaaSqaamaabmaabaGaemiCaaNaeiilaWIaemizaqgacaGLOaGaayzkaaaabeaakiabcIcaOiabdgeabnaaBaaaleaadaqadaqaaiabdchaWjabcYcaSiabdsgaKbGaayjkaiaawMcaaaqabaGccqGGPaqkaSqaamaabmaabaGaemiCaaNaeiilaWIaemizaqgacaGLOaGaayzkaaGaeiilaWIaemyqae0aaSbaaWqaamaabmaabaGaemiCaaNaeiilaWIaemizaqgacaGLOaGaayzkaaaabeaaliabc6ha+naabmaabaGaemiCaaNaeiilaWIaemizaqgacaGLOaGaayzkaaaabeqdcqGHpis1aOGaeyyXIC9aaebuaeaacqWFgpGzdaWgaaWcbaGae8hWdahabeaakmaabmaabaGaemiwaG1aaSbaaSqaaiab=b8aWbqabaGccqGGSaalcqWGtbWudaWgaaWcbaGae8hWdahabeaakiabcYcaSiabdggaHnaaBaaaleaacqWFapaCaeqaaOGaei4oaSJaemiCaaNaeiilaWIaemizaqgacaGLOaGaayzkaaaaleaacqWFapaCcqGHiiIZcqqHGoaudaWgaaadbaGaeiikaGIaemiCaaNaeiilaWIaemizaqMaeiykaKcabeaaaSqab0Gaey4dIunakiabgwSixpaarafabaGae8NXdy2aaSbaaSqaaiabd+gaVjabdchaWbqabaGcdaqadiqaaiabdgeabnaaBaaaleaacqWGVbWBcqWGWbaCaeqaaaGccaGLOaGaayzkaaaaleaacqWGbbqqdaWgaaadbaGaem4Ba8MaemiCaahabeaaaSqab0Gaey4dIunakiabc6caUiaaxMaacaWLjaGaeiikaGIaeGOnayJaeiykaKcaaa@9740@

where *D *is the set of perturbation tuples (*p*, *d*), *A*_(*p*, *d*) _the activities of valid paths connecting (*p*, *d*), and *A*_*op *_the activities of paths subject to the same operon constraint.

### Inferring the optimal configurations

We want to find the attribute values of the entire model (MAP configurations) which maximize the joint likelihood function in equation 6. This is a standard inference problem in graphical models and can be approximately solved by recursively applying the max-product algorithm of factor graphs. Details about the max-product algorithm and a recursive algorithm for finding the optimal configurations can be found in [25] and [29].

### Explaining and predicting perturbation data

To evaluate the explanatory power of the model we compare the predicted responses according to the inferred MAP configurations to the observed perturbation data. Given a configuration, for each (perturbation, response) tuple we identify the active paths connecting the tuple according to the configuration. We then predict the response along each active path using the procedure described above. A (perturbation, response) tuple is explained by a configuration if the predicted responses along all active paths are identical to the actual response. A (perturbation, response) tuple is explained by the model if it is explained by all MAP configurations inferred from the model. In contrast, a (perturbation, response) tuple is contradicted with the model if the model has a consistent prediction but the consistent prediction is the opposite of the observed response. We thereby evaluate the explanatory power of the model by counting the number of (perturbation, response) tuples explained by the model minus the number of (perturbation, response) tuples contradicted with the model.

The same procedure is also used to evaluate the predictive power of the model in a cross-validation setting. The MAP configurations are inferred from the training data and we check whether the predicted responses along all active paths in each MAP configuration are consistent with the test data. Different methods of splitting the training and test data are described in Results.

We apply permutation tests to further justify the significance of the model's explanatory power. The perturbation responses of genes or fluxes in each experiment are randomly permuted. The numbers of permuted (perturbation, response) tuples consistent or contradicted with model prediction are counted. The p-value of the model's explanatory power is the fraction of random trials whose (# explained tuples – # contradicted tuples) exceed the empirical value.

### Identifying the coupling between metabolic and regulatory networks

We propose two methods to identify and test the coupling between gene regulation and metabolic reactions. The first approach attempts to learn the feedback links between metabolites and transcription factors from the perturbation data. We identify those missing links in terms of their power to explain the perturbation data. Define the explanatory power of a set of (metabolite, regulator) links as the number of explained perturbation responses minus the number of contradicted perturbation responses of the joint network model with those links. Our goal is to find the links which maximize the explanatory power. We apply a simple method of incrementally adding (metabolite, regulator) links which maximize the gain of the explanatory power. Candidate links are the pairs between all metabolites and all regulators. At each iteration we find the link which yields the maximum gain of the explanatory power. When multiple links possess equal explanatory power, we branch the search process by creating multiple joint networks, each with one optimal link added. Edge addition stops when the gain of the explanatory power is statistically insignificant according to the permutation test (*p *> 10^-3^).

The second approach explicitly tests abstract "design rules" pertaining to the transcriptional regulation of enzymes. A general hypothesis is encoded in the model by adding links from metabolites to enzyme genes and designating their functions (edge signs). For example, to encode hypothesis 4 (glucose repression) we add negative edges from glucose to enzymes in TCA cycle and genes involved in electron transfer. A factor graph model is then built on the augmented joint network to explain the perturbation data.

### Implementation information

We encoded the programs of model inference, search and validation in C, and ran the programs on a dual-processor Linux PC (Intel Pentium 4 CPU, 3 GHz, Red Hat Linux 3.4.4, 1 GB memory). The running time for inferring the optimal configurations given the fixed links or general hypotheses varies with the maximum path length. For the maximum path length equal to 6, the running time is less than 10 seconds. The running time for searching the optimal links among all (metabolite, regulator) pairs with maximum path length 6 is about 30 minutes. The running time for leave-one-out, 5-fold or 10-fold cross validation with maximum path length 6 is about 30 minutes. The source codes of these programs and the tabulated forms of the perturbation data are provided in the supplementary files [see Additional files 15, 16].

## Authors' contributions

CHY and MV conceived the models. CHY implemented the models, collected and analyzed data with the models, and wrote the paper.

## Supplementary Material

Additional file 1 Active paths explaining expression changes in glucose reduction, model of inferred links. Click here for file

Additional file 2 Active paths explaining expression changes in pykF knock-out, model of inferred links. Click here for file

Additional file 3 Active paths explaining expression changes in acetate enhancement, model of inferred links. Click here for file

Additional file 4 Active paths explaining flux changes in pykA/pykF double knock-outs, model of inferred links. Click here for file

Additional file 5 Active paths explaining flux changes in pgi knock-out, model of inferred links. Click here for file

Additional file 6 Active paths explaining flux changes in zwf knock-out, model of inferred links. Click here for file

Additional file 7 Active paths explaining expression changes in glucose reduction, model of general hypotheses. Click here for file

Additional file 8 Active paths explaining expression changes in pykF knock-out, model of general hypotheses. Click here for file

Additional file 9 Active paths explaining expression changes in acetate enhancement, model of general hypotheses. Click here for file

Additional file 10 Active paths explaining flux changes in pykA/pykF double knock-outs, model of general hypotheses. Click here for file

Additional file 11 Active paths explaining flux changes in pgi knock-out, model of general hypotheses. Click here for file

Additional file 12 Active paths explaining flux changes in zwf knock-out, model of general hypotheses. Click here for file

Additional file 13 The number of perturbation responses explained and contradicted by the model of inferred links with varying length, categorized by (1)types of responses (2)magnitudes of responses (3)types of paths explaining the responses. Click here for file

Additional file 14 The number of perturbation responses explained and contradicted by the model of general hypotheses with varying length, categorized by (l)types of responses (2)magnitudes of responses (3)types of paths explaining the responses. Click here for file

Additional file 15 The source codes of the model inference programs. Click here for file

Additional file 16 The data of the *E. coli *central carbon network and perturbation experiments. Click here for file

## Figures and Tables

**Table 1:** Perturbation data used in this work

Source	Response	Reference
acetate ↑	expression	Oh et al., 2000
glucose ↓ vs amino acid ↓	expression	Hua et al., 2004
pykF ↓	expression	Siddiquee et al., 2004
Pgi ↓	flux	Hua et al., 2003
pykA ↓ pykF ↓	flux	Emmerling et al., 2002
zwf ↓	flux	Hua et al., 2003

**Table 2:** (metabolite, regulator) sets inferred from the model.

length	set	net explained	pval
1		1	1.0
2		1	1.0
3	(glucose, ArcA) (acetate, ArcA)	34	< 10^-5^
4	(g6p, ArcA) (acetyl-CoA, ArcA)	38	< 10^-5^
4	(g6p, ArcA) (acetylphosphate, ArcA)	38	< 10^-5^
4	(g6p, ArcA) (acetate, ArcA)	38	< 10^-5^
4	(g6p, ArcA) (pyrophosphate, ArcA)	38	< 10^-5^
5	(g6p, ArcA) (pyruvate, ArcA)	65	< 10^-5^
6	(g6p, ArcA) (pyruvate, ArcA)	66	< 10^-5^
6	(g6p, ArcA) (acetyl-CoA, ArcA)	66	< 10^-5^
6	(g6p, ArcA) (malate, ArcA)	66	< 10^-5^
6	(g6p, ArcA) (2k3d6pg, ArcA)	66	< 10^-5^
7	(g6p, ArcA) (pyruvate, ArcA) (pepyruvate, FruR)	74	< 10^-5^
8	(f6p, ArcA) (pepyruvate, ArcA) (2pg, FruR)	78	< 10^-5^
9	(f6p, ArcA) (pepyruvate, ArcA) (6pg, FruR)	72	< 10^-5^

The explanatory power (net explained) is (# explained responses – # contradicted responses). The p-value of the explanatory power is calculated by randomly permuting the perturbation responses over genes or fluxes. No feedback links are added to the network when the maximum path length ≤ 2, since short paths containing feedback links can not explain the data. Abbreviation of metabolites: g6p: glucose-6-phosphate, pepyruvate: phosphoenolpyruvate, f6p: fructose-6-phosphate, 6pg: 6-phospho-gluconate, 2pg: 2-phosphoglycerate, 2k3d6pg: 2-keto-3-deoxy-6-phospho-gluconate.

**Table 3:** Explanatory power and significance of models (a) inferred links (b) general hypotheses

length		1	2	3	4	5	6	7	8	9
total	explained-contradict	1	1	27	38	44	67	70	70	72
	pval	1.0	1.0	< 1.0·10^-5^	< 1.0·10^-5^	< 1.0·10^-5^	< 1.0·10^-5^	< 10^-5^	< 10^-5^	< 10^-5^
mixed	explained-contradict	1	1	21	27	27	45	41	41	41
	pval	1.0	1.0	1.1·10^-3^	< 1.0·10^-5^	< 1.0·10^-5^	< 1.0·10^-5^	< 10^-5^	< 10^-5^	< 10^-5^

length		1	2	3	4	5	6	7	8	9

total	explained-contradict	1	1	28	40	49	58	63	75	75
	pval	1.0	1.0	< 1.0·10^-5^	1.0·< 10^-5^	< 1.0·10^-5^	< 1.0·10^-5^	< 10^-5^	< 10^-5^	< 10^-5^
mixed	explained-contradict	1	1	21	29	32	36	34	44	44
	pval	1.0	1.0	2.5·10^-3^	1.34·10^-3^	9.1·10^-4^	1.6·10^-4^	1.9·10^-4^	< 10^-5^	< 10^-5^
